# LFuji-air dataset: Annotated 3D LiDAR point clouds of Fuji apple trees for fruit detection scanned under different forced air flow conditions

**DOI:** 10.1016/j.dib.2020.105248

**Published:** 2020-02-07

**Authors:** Jordi Gené-Mola, Eduard Gregorio, Fernando Auat Cheein, Javier Guevara, Jordi Llorens, Ricardo Sanz-Cortiella, Alexandre Escolà, Joan R. Rosell-Polo

**Affiliations:** a Research Group in AgroICT & Precision Agriculture, Department of Agricultural and Forest Engineering, Universitat de Lleida (UdL) – Agrotecnio Center, Lleida, Catalonia, Spain; b Department of Electronic Engineering, Universidad Técnica Federico Santa María, Valparaíso, Chile

**Keywords:** Fruit detection, Fruit location, Yield prediction, LiDAR, MTLS, Fruit reflectance, Forced air flow

## Abstract

This article presents the LFuji-air dataset, which contains LiDAR based point clouds of 11 Fuji apples trees and the corresponding apples location ground truth. A mobile terrestrial laser scanner (MTLS) comprised of a LiDAR sensor and a real-time kinematics global navigation satellite system was used to acquire the data. The MTLS was mounted on an air-assisted sprayer used to generate different air flow conditions. A total of 8 scans per tree were performed, including scans from different LiDAR sensor positions (multi-view approach) and under different air flow conditions. These variability of the scanning conditions allows to use the LFuji-air dataset not only for training and testing new fruit detection algorithms, but also to study the usefulness of the multi-view approach and the application of forced air flow to reduce the number of fruit occlusions. The data provided in this article is related to the research article entitled “Fruit detection, yield prediction and canopy geometric characterization using LiDAR with forced air flow” [1].

**Table:** Specifications Table

Subject	Agronomy and Crop Science, Horticulture, Computer Vision and Pattern Recognition
Specific subject area	Precision Agriculture, Fruit Detection
Type of data	LiDAR based point clouds Fruit location annotations
How data were acquired	Data was acquired with a Mobile Terrestrial Laser Scanner (MTLS) comprised of a LiDAR Sensor and a real-time kinematics global navigation satellite system (RTK-GNSS).
Data format	Raw LiDAR data: *PCAP* Raw RTK-GNSS data: *TXT* 3D point clouds: *MAT* Annotations: *TXT*
Parameters for data collection	The MTLS forward speed was set to 0.125 m s^−1^. The LiDAR sensor acquired data at a frequency of 10 Hz, while the RTK-GNSS sensor provided positioning measurements with a precision of ±0.01/0.02 m (horizontal/vertical) at 20 Hz frequency rate. The system was mounted on an air-assisted sprayer which generated an air flow speed of 5.5 ± 2.3 m s^−1^ (measured at 2.4 m from the sprayer fan).
Description of data collection	A MTLS was used to scan 11 Fuji apple trees containing a total of 1444 apples. The MTLS was mounted on an air-assisted sprayer used to generate different air flow conditions. A total of 8 different scanning conditions were tested, corresponding to the following combinations: two different sensor positions (1.8 m and 2.5 m height); two different air flow conditions (sprayer fan switched on and off); scans from the two sides of the row of trees (East and West). The ground truth of the apples locations was manually generated by placing 3D rectangular bounding boxes around each apple position.
Data source location	City/Town/Region: *Agramunt, Catalonia* Country: *Spain* GPS coordinates for collected data: *E: 336297 m, N: 4623494 m, 312 m a.s.l., UTM 31T - ETRS89*
Data accessibility	Repository name: *GRAP datasets/Lfuji-air dataset* Data identification: *Lfuji-air dataset* Direct URL to data: http://www.grap.udl.cat/en/publications/datasets.html
Related research article	[1] Gené-Mola J, Gregorio E, Auat Cheein F, Guevara J, Llorens J, Sanz-Cortiella R, Escolà A, Rosell-Polo JR. Fruit detection, yield prediction and canopy geometric characterization using LiDAR with forced air flow Submitted in *Computers and Electronics in Agriculture*

**Table:** 

**Value of the Data** • First dataset for fruit detection containing annotated LiDAR based 3D data acquired from different sensor positions and under different air flow conditions. • The dataset allows testing fruit detection algorithms based on LiDAR based 3D data. • Precision horticulture community can benefit from these data to test methodologies with applications in yield prediction, yield mapping and canopy geometric characterization. • Presented data can be used for analysing the effect of applying forced air flow and multi-view sensing for reducing the number of occlusions in fruit detection

## 1 Data description

### 1.1 Data repository

**Fig. 1:**
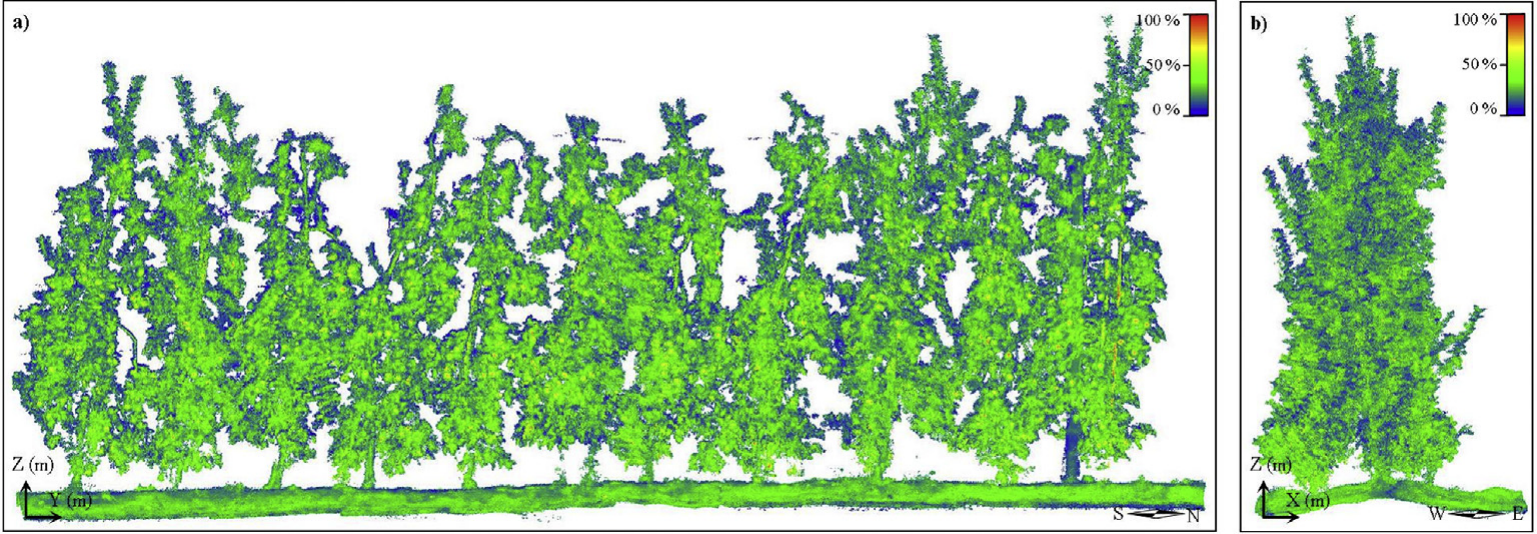
3D point cloud of all trees included in the dataset (11 trees). a) Front elevation view. b) Left side elevation view. The color scale illustrates the points reflectance, ranging from 0% (blue) to 100% (red). *X*, *Y*, and *Z* indicate the direction of the global axis, while *N*, *S, E,* and *W* represent the cardinal directions.

The repository *Lfuji-air dataset* (http://www.grap.udl.cat/en/publications/LFuji_air_dataset.html) includes 3D LiDAR point clouds of 11 Fuji apple trees (*Malus domestica* Borkh. Cv. Fuji) containing 1444 apples (Fig. 1). A total of 8 point clouds are provided for each tree, corresponding to the combinations of the following scanning conditions:

• *H1*: LiDAR sensor positioned at the height of 1.8 m from the floor. This LiDAR position corresponds (approximately) to the half of the tree heights.
• *H2*: LiDAR sensor positioned at the height of 2.5 m from the floor.
• *n*: Trees scanned without forced air flow application.
• *af*: Trees scanned under forced air flow conditions.
• *E*: Data acquired from the East side of the row of trees.
• *W*: Data acquired from the West side of the row of trees.

Point clouds were saved in MAT format. Each MAT file contains the data from one tree *#T#* (*01–11*), scanned with the LiDAR sensor at height *#H#* (*H1* or *H2*), under air flow conditions *#F#* (*n* or *af*), and from the side *#S#* (*E* or *W*). From that, the point clouds files are named as “Tree*#T#*_*#H#*_*#F#*_*#S#*.mat”. For instance, the file “Tree07_H2_af_E.mat” contains the point cloud of Tree 7, obtained with the LiDAR sensor at height 2.5 m (H2), by applying forced air flow (af), and scanned from the east (E) side. Data inside the MAT files is organized in an m*4 matrix, where the three first columns give the position of the points in global world coordinates ([*X, Y, Z*]_<*Global*>_), and last column corresponds to each point reflectance (*R*).

The dataset includes a total of 1353 apple annotations (out of 1444 apples manually counted in field). The remaining 6.3% apples could not be identified in the point cloud because they were not visible (from a human/visual inspection). Annotations are provided in TXT format, where the first row indicates the position of the apple centre, while the following eight rows correspond to the positions of the bounding box corners.

Raw data used to generate the 3D point clouds is also provided in the dataset. This includes LiDAR data in *PCAP* format, and the positions of the real-time kinematics global navigation satellite system (RTK-GNSS) system in *TXT* format. Section 2 describes how raw data was acquired and processed to generate the described point clouds.

### 1.2 Code repository

The code used to process the row data and generate the georeferenced point clouds has been made publicly available at https://github.com/GRAP-UdL-AT/MTLS_point_cloud_generation. This Matlab code combines the LiDAR and RTK-GNSS raw data to obtain the 3D model of the measured trees. Section 2.3 describes the transformation matrices implemented in this code.

Additionally, the code used in Ref. [1] for fruit detection using the present dataset has also been made publicly available at https://github.com/GRAP-UdL-AT/fruit_detection_in_LiDAR_pointClouds. This code was developed to train and test the fruit detection algorithm as well as studying different sensor heights and air flow conditions to reduce the number of fruit occlusions. Both processing codes presented in this section were implemented using MATLAB® (R2018a, Math Works Inc., Natick, Massachusetts, USA).

## 2 Materials and methods

### 2.1 Experimental design

**Fig. 2:**
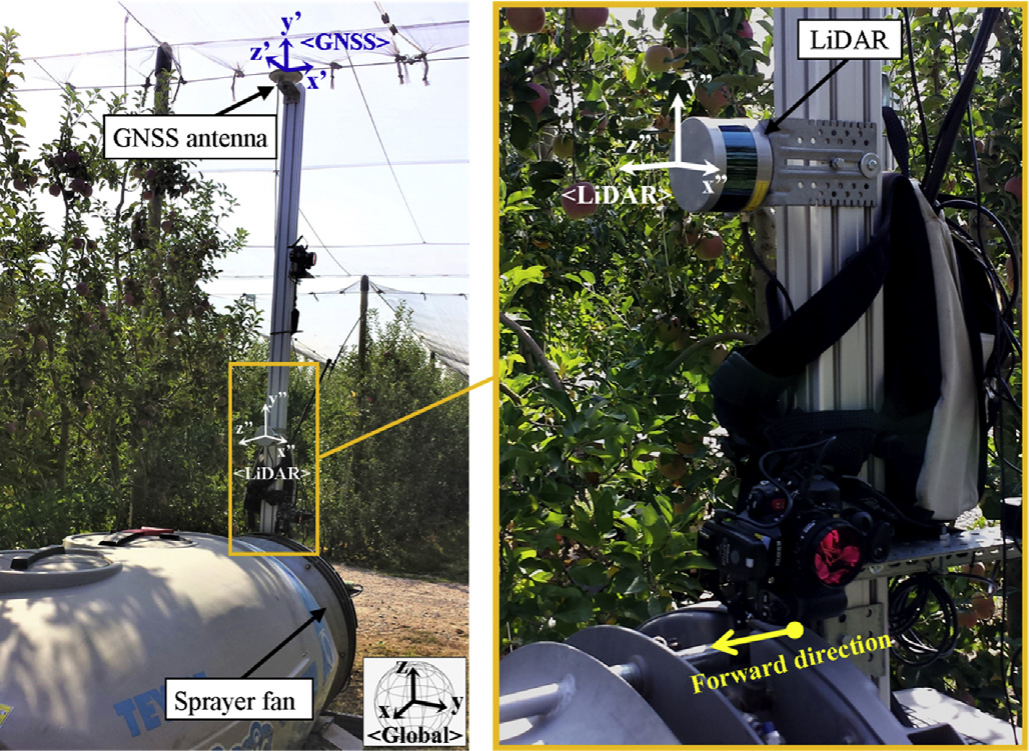
Global scheme of the experimental setup used for data acquisition. The orientation of <LiDAR>, <GNSS>, and <Global> coordinate systems used for point cloud generation are represented.

Data was collected in a commercial Fuji apple orchard (*Malus domestica* Borkh. cv. Fuji). A total of 11 consecutive trees containing 1444 apples were scanned 3 weeks before harvesting, at 85 BBCH growth stage [2]. The experimental setup used for data acquisition was a mobile terrestrial laser scanner (MTLS) comprised of a LiDAR sensor and a RTK-GNSS (Fig. 2). Both sensors were connected to a rugged laptop used to acquire and synchronise the data by means of the acquisition time.

The LiDAR sensor was a Puck VLP-16 (Velodyne LIDAR Inc., San José, CA, USA), which generates a 3D point cloud of the scanned scene in the <*LiDAR*> coordinate system (Fig. 2) with an accuracy of ±0.03 m (typical) at a frequency of 10 Hz (manually set). Additionally, this sensor provides the calibrated reflectance of each point (*R*) [3], which is a valuable information for fruit detection due to the different reflectance of apples and background [4]. The RTK-GNSS system used was a GPS1200+ (Leica Geosystems AG, Heerbrugg, Switzerland), which provides position measurements of the MTLS in <*Global*> world coordinates (Fig. 2) at a frequency of 20 Hz with an absolute error of 0.01/0.02 m (horizontal/vertical). Further specifications of the LiDAR and RTK-GNSS sensors used are detailed in Table 1.

**Table 1:** Mobile terrestrial laser scanner set up specifications.

LiDAR sensor	Manufacturer and model	Velodyne Puck VLP-16
	Number of laser beams	16
	Measurement Range	100 m
	Measurement accuracy	±30 mm
	Field of View (Horizontal//Vertical)	30°//150° (manually set)
	Angular Resolution (Horizontal//Vertical)	2.0°//0.2°
	Scan Rate	10 Hz (manually set)
	Wavelength	903 nm
RTK-GNSS	Manufacturer and model	Leica GPS1200+
	Measurement accuracy	20 mm
	Measurement Rate	20 Hz

The MTLS system was mounted on an air-assisted sprayer, next to the sprayer fan, which was used to generate forced air flow and move the tree foliage. The GNSS antenna was installed at a height of 3.5 m. The LiDAR sensor was mounted vertically, with the *Z*_<*LiDAR*>_ axis pointing to the forward direction (Fig. 2), and placed at heights of 1.8 m (*H1*) and 2.5 m (*H2*). The experimental setup was pulled by a tractor at 0.125 m s^−1^ forward speed and following a linear trajectory parallel to the row of trees.

### 2.2 Sprayer fan characterization

**Fig. 3:**
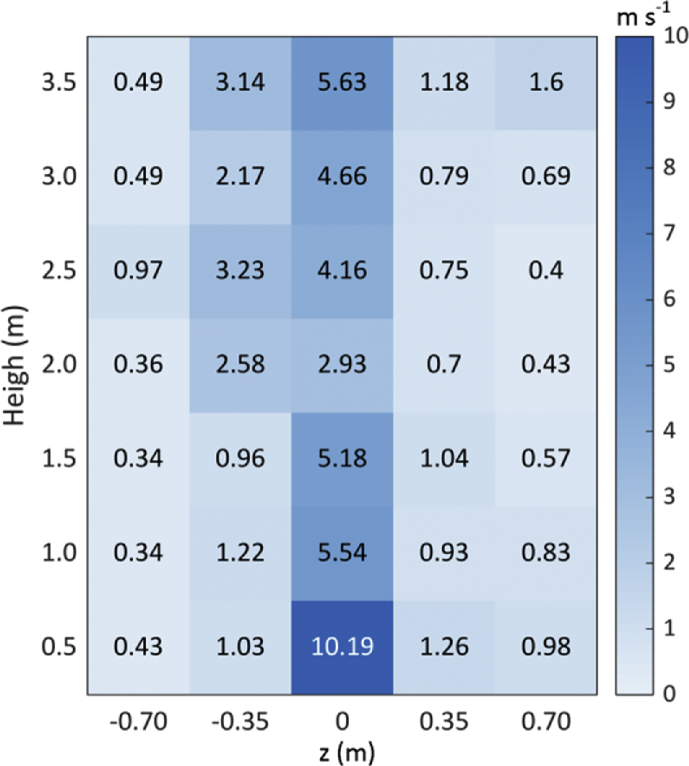
Air flow speed in *m s*^*−1*^ at different heights and widths (z) measured at a distance of 2.4 m from the sprayer fan.

In order to generate forced air flow, the air-assisted sprayer operated at 18π rad s^−1^ (540 rpm of PTO, power take-off angular speed). At these conditions, the air flow speed at different heights and widths was characterized using an AIRMAR 200WX weather station (AIRMAR Technology Corporation, Milford, NH, USA), which measures the wind speed with an accuracy of ±0.5 m s^−1^. A total of 35 measurements from a distance of 2.4 m (distance between sprayer fan and scanned trees) were performed, corresponding to the measurement of 7 height and 5 width intervals (Fig. 3). The 7 height intervals were equally distributed from 0 m to 3.5 m height, corresponding to the maximum trees height. On the other hand, the 5 width intervals were equally distributed along 1.4 m width, which corresponds to the field-of-view of the LiDAR sensor. The speed values shown in Fig. 3 are the result of averaging 10 measurements in each position.

### 2.3 Point cloud generation

The LiDAR row data consist on a set of frames acquired from different positions, where each frame $P_{<LiDAR>}$ is a point cloud in the <*LiDAR*> coordinate system:

(1) $$P_{\langle LiDAR\rangle }=\left[\begin{array}{ccc} \begin{array}{c} x_{1}\\ y_{1}\\ \begin{array}{c} z_{1}\\ 1 \end{array} \end{array} & \begin{array}{c} x_{2}\\ y_{2}\\ \begin{array}{c} z_{2}\\ 1 \end{array} \end{array} & \begin{array}{cc} \begin{array}{c} \ldots \\ \ldots \\ \begin{array}{c} \ldots \\ \ldots \end{array} \end{array} & \begin{array}{c} x_{n}\\ y_{n}\\ \begin{array}{c} z_{n}\\ 1 \end{array} \end{array} \end{array} \end{array}\right]$$

where n denotes the number of points in the LiDAR frame.

For the generation of the 3D point cloud of all trees (Fig. 1), each frame $P_{<LiDAR>}$ was transformed into <*Global*> coordinates as follows:

(2) $$P_{\langle Global\rangle }=T_{\langle LiDAR\rangle \;\;\to \;\;\langle Global\rangle }\times P_{\langle LiDAR\rangle }$$

where the transformation matrix $T_{<LiDAR>\to <Global>}$ can be expanded as:

(3) $$T_{\langle LiDAR\rangle \;\;\to \;\;\langle Global\rangle }=T_{\langle GNSS\rangle \;\;\to \;\;\langle Global\rangle }\times T_{\langle LiDAR\rangle \;\;\to \;\;\langle GNSS\rangle }$$

Because the LiDAR and the GNSS antenna were assembled in a rigid structure, the rigid transformation matrix $T_{<LiDAR>\;\;\to \;\;<GNSS>}$ only has a translational offset:

(4) $$T_{\langle LiDAR\rangle \;\;\to \;\;\langle GNSS\rangle }=I|\Delta xyz_{H1\langle LiDAR\rangle \langle GNSS\rangle }=\left[\begin{array}{ccc} \begin{array}{c} 1\\ 0\\ \begin{array}{c} 0\\ 0 \end{array} \end{array} & \begin{array}{c} 0\\ 1\\ \begin{array}{c} 0\\ 0 \end{array} \end{array} & \begin{array}{cc} \begin{array}{c} 0\\ 0\\ \begin{array}{c} 1\\ 0 \end{array} \end{array} & \begin{array}{c} \Delta x_{\langle LiDAR\rangle \langle GNSS\rangle >}\\ \Delta y_{\langle LiDAR\rangle \langle GNSS\rangle }\\ \begin{array}{c} \Delta z_{\langle LiDAR\rangle \langle GNSS\rangle }\\ 1 \end{array} \end{array} \end{array} \end{array}\right]$$

where τ=[Δx<LiDAR><GNSS>, Δy<LiDAR><GNSS>,Δz<LiDAR><GNSS>]'   denotes the offset between each axis of the GNSS and the LiDAR sensor. Considering the distribution of the sensors in the experimental setup, the translation offsets for the *H1* and *H2* trials were τ=[0,1.768,0.058]'m and τ=[0,1.07,0.058]'m, respectively.

Meanwhile, the rigid transformation matrix $T_{<GNSS>\;\;\to \;\;<Global>}$ includes a rotational, $R_{<GNSS>}$, and a translational, $T_{<GNSS>}$, component. As depicted in Fig. 2, the forward direction is $z{\text{'}}_{<GNSS>}$. Being θ and φ the orientation angles of the vehicle around the $y{\text{'}}_{<GNSS>}$ (Yaw) and $x{\text{'}}_{<GNSS>}$ (Pitch) axes, respectively, the transformation matrix $T_{<GNSS>\;\;\to \;\;<Global>}$ is obtained according to:

(5) $$T_{\langle GNSS\rangle \to \langle Global\rangle }=R_{\langle GNSS\rangle }|T_{\langle GNSS\rangle }=\left[\begin{array}{ccc} \begin{array}{c} \cos\;\theta \\ \sin\;\theta \\ \begin{array}{c} 0\\ 0 \end{array} \end{array} & \begin{array}{c} -\sin\;\theta \;\cos\;\varphi \\ \cos\;\theta \;\cos\;\varphi \\ \begin{array}{c} \sin\;\varphi \\ 0 \end{array} \end{array} & \begin{array}{cc} \begin{array}{c} \sin\;\theta \;\sin\;\varphi \\ -\cos\;\theta \;\sin\;\varphi \\ \begin{array}{c} \cos\;\varphi \\ 0 \end{array} \end{array} & \begin{array}{c} X_{\langle GNSS\rangle }\\ Y_{\langle GNSS\rangle }\\ \begin{array}{c} Z_{\langle GNSS\rangle }\\ 1 \end{array} \end{array} \end{array} \end{array}\right]$$

Where $\;X_{<GNSS>},\;Y_{<GNSS>}\;$ and $Z_{<GNSS>}$ denote the position of the GNSS antenna in each axis of the global coordinate system. It is worth to mention that the experimental setup did not include an inertial measurement unit (IMU); therefore, the orientation angles θ y φ were obtained by using the forward direction computed from the measurements of the RTK-GNSS receiver. Since trials were conducted in short rectilinear trajectories, the orientation of the system was assumed to be constant along the path.

The resulting point clouds were manually split into a single point cloud per tree. Then each tree was manually annotated by placing 3D rectangular bounding boxes around each apple position. This process was carried out using the software CloudCompare (Cloud Compare [GPL software] v.9 Omnia) and supported by additional RGB images of the tested trees.
